# Multiplexed Detection of *O*-GlcNAcome, Phosphoproteome, and Whole Proteome within the Same Gel

**DOI:** 10.3389/fendo.2014.00184

**Published:** 2014-10-28

**Authors:** Caroline Cieniewski-Bernard, Erwan Dupont, Barbara Deracinois, Matthias Lambert, Bruno Bastide

**Affiliations:** ^1^Université Lille Nord de France, Lille, France; ^2^EA4488, APMS (Physical activity muscle and health), URePSSS, Université de Lille 1, Villeneuve d’Ascq, France

**Keywords:** O-GlcNAcylation, phosphorylation, proteomic analysis, 2D-electrophoresis, click-chemistry, multiplexed proteomics technology, fluorescent dyes

## Abstract

The cellular diversity of proteins results in part from their post-translational modifications. Among all of them, the O-GlcNAcylation is an atypical glycosylation, more similar to phosphorylation than classical glycosylations. Highly dynamic, reversible, and exclusively localized on cytosolic, nuclear, and mitochondrial proteins, O-GlcNAcylation is known to regulate almost all if not all cellular processes. Fundamental for the cell life, O-GlcNAcylation abnormalities are involved in the etiology of several inherited diseases. Assessing to O-GlcNAcylation pattern will permit to get relevant data about the role of O-GlcNAcylation in cell physiology. To get understanding about the role of O-GlcNAcylation, as also considering its interplay with phosphorylation, the *O*-GlcNAc profiling remains a real challenge for the community of proteomists/glycoproteomists. The development of multiplexed proteomics based on fluorescent detection of proteins permits to go further in the understanding of the proteome complexity. We propose herein a multiplexed proteomic strategy to detect *O*-GlcNAcylated proteins, phosphoproteins, and the whole proteome within the same bidimensional gel. In particular, we investigated the phosphoproteome through the ProQ Diamond staining, while the whole proteome was visualized through Sypro Ruby staining, or after the labeling of proteins with a T-Dye fluorophore. The *O*-GlcNAcome was revealed by the way of the Click chemistry and the azide–alkyne cycloaddition of a fluorophore on GlcNAc moieties. This method permits, after sequential image acquisition, the direct in-gel detection of *O*-GlcNAcome, phosphoproteome, and whole proteome.

## Background

O-GlcNAcylation got 30 years old, the “age of reason.” Since its discovery in 1984 by Gerald W. Hart (1, 2), O-GlcNAcylation was demonstrated by turn to be involved in numerous cellular processes, in particular, transcription, translation, signal transduction, proteasomal degradation, cellular stress, and so on (3–5). Nowadays, no one could refuse that O-GlcNAcylation is a key modulator in almost all if not all cellular processes. Furthermore, a dysregulation of O-GlcNAcylation cycling is associated to the physiopathology of several acquired diseases, such as cancers, type 2 diabetes, neurodegeneration, or cardiovascular disorders (6–9). The *O*-GlcNAc profiling, assessing to O-GlcNAcylation pattern and the quantification of variation of O-GlcNAcylation on proteins, remains an important challenge for the understanding of the role of this atypical glycosylation on the regulation of cellular processes or on the physiopathology of several inherited diseases.

Several antibodies directed against *O*-GlcNAc moieties are currently available [detailed in Ref. (5, 10)], enhancing greatly the probe and/or the enrichment of *O*-GlcNAcylated proteins. Classical methods of quantification of O-GlcNAcylation variation on given proteins are based on immunoprecipitation coupled to western blot analysis. Thus, upstream enrichment of *O*-GlcNAc bearing-proteins (through immunoprecipitation or lectin enrichment) followed by antibody-based detection of a protein of interest through western blot analysis remains a common practice to quantify relative changes of *O*-GlcNAc level on a target protein. This method remains a suitable tool for “oriented” investigation about the role of O-GlcNAcylation in a given cellular pathway. However, while largely and routinely used in laboratories, this classical approach suffers from an important limitation due to the selection of the proteins of interest by the researchers: only a slight number of proteins could be considered.

Furthermore, it is well-admitted that O-GlcNAcylation could not be considered alone, because of its dynamic interplay with phosphorylation (4, 11–13). This interplay could be investigated using the approach described above with minor changes, the antibody used in western blot being directed against the phospho-epitope of the protein. However, while many phospho-specific antibodies are currently available (for example, those directed against proteins from key intracellular processes), several proteins known to be phosphorylated suffer from the lack of a specific antibody directed against their phosphorylated epitope. Thus, we have recently coupled this immunoprecipitation/western blot methodology with the Phos-Tag electrophoresis to quantify the variation of O-GlcNAcylation on proteins separated according to their phosphorylation status (14). Interestingly, using a Phos-Tag acrylamide incorporated directly into the monodimensional gel, different states of a given protein could be separated according to the number of phosphate moieties and the variation of O-GlcNAcylation could be determined on each phosphorylated form of the protein.

Indeed, to gain in understanding of O-GlcNAcylation dynamics occurring during cell or tissue status changes, proteomic analysis remains a method of choice to undergo changes in glycosylation level of proteins. Despite the fact of intrinsic limitations of 2D-electrophoresis (analysis of membrane proteins, divergent proteins expression in cells or tissues, high-chemical diversity of proteins …) (15), bidimensional electrophoresis enables to get relevant information through the cartography of the proteome at a given time and under particular physiological conditions, and is a powerful strategy to characterize multiple modified proteins (16). The consideration of *O*-GlcNAcome map was recently successfully investigated using a gel-based strategy. Proteins were separated on 2D-gels and were transferred on membrane for detection of *O*-GlcNAc moieties using CTD110.6 or RL-2 antibodies or lectins, leading to the identification of *O*-GlcNAcylated proteins, and/or those presenting a modulation in their O-GlcNAc level (17–20). Based on western blot analysis, this kind of approach could be coupled to detection of phosphoproteome using antibodies directed against the phospho-amino acids (21). One of the major difficulties in this kind of approach is the alignment between the 2D-western blot and Coomassie- or silver-stained 2D-gels using images software. To avoid this difficulty, fluorescent detection of proteins in gels is gaining popularity and large-scale use since it gains in reproducibility and its linear dynamic range of detection. In this way, the Van Eyk group’s assessed the N-linked and O-GlcNAcylation in human and simian immunodeficiency viruses using a 2D-gel approach, and a detection of glycosylated proteins using the ProQ Emerald staining (22). In a previous study, the use of this fluorescent dye was coupled to ProQ Diamond staining in order to detect glycosylated and phosphorylated forms of proteins, the whole proteins pattern being detected using Sypro Ruby staining (23).

We propose herein a multiplexed proteomic strategy to detect *O*-GlcNAcylated proteins, phosphoproteins, and the whole proteome within the same gel. Detection of *O*-GlcNAcylated proteins was done after labeling of sugar moieties by a fluorophore (TAMRA or Alexa Fluor^®^ 488), the (3 + 2) azide–alkyne cycloaddition of the fluorophore required the preliminary incorporation of an azide function on the *O*-GlcNAcylated moieties. We compared the metabolic incorporation of GlcNAz (azido-modified *N*-acetylglucosamine) moieties and the labeling of *O*-GlcNAcylated proteins with GalNAz (azido-modified *N*-acetylgalactosamine) through the engineered β-1,4-galactosyltransferase (Y289L GalT). While the detection of proteins phosphorylated on serine, threonine, and tyrosine residues was previously performed with success, in particular, in view of a bottom-up proteomic strategy (24–26), we detected the phosphoproteome using the ProQ Diamond dye. Finally, the global proteome was detected through the fluorescent dye Sypro Ruby, as it was described in classical multiplexed approaches, or after labeling of proteins using the T-Dye. The sequential image acquisitions permitted, from only one gel, a direct visualization of *O*-GlcNAcylated proteins, phosphorylated proteins, and the whole proteins pattern.

## Detailed Experimental Procedures

### C2C12 cell culture

#### Proliferation and differentiation of C2C12 cells

The C2C12 mouse myoblasts were obtained from American Type Culture Collection (ATCC, Manassas, VA, USA). C2C12 myoblasts were grown to 80–90% confluence in Dubelcco’s Modified Eagle Medium (Gibco) supplemented with 10% fetal calf serum (Gibco) and 1% antibiotics/antimycotics, at 37°C in a 5% CO_2_-humidified atmosphere. They were then induced to differentiate into myotubes by switching to DMEM containing 2% heat-inactivated horse serum (differentiation medium, DM). The shifting time to DM was assigned to day 0 of differentiation. Media were changed every 48 h, and myotubes formation was monitored daily. All experiments were performed on 5-day differentiated myotubes, this state of differentiation, corresponded to mature myotubes, was chosen according to preliminary experiments.

#### Metabolic labeling

In certain experiments, 50 μM of Ac_4_-GlcNAz (tetraacetylated *N*-azidoacetylglucosamine, Molecular Probes), diluted in DMSO, was added in cell medium for 48 h. Control condition corresponded to cells incubated with DMSO alone (vehicle condition). It is noteworthy that we evaluated the efficiency of the metabolic incorporation of Ac_4_-GlcNAz after coupling the GlcNAz moieties to Alexa Fluor^®^ 488 fluorophore, as well as cell viability. Several concentrations of Ac_4_-GlcNAz and treatment durations were tested.

#### Cell viability test

C2C12 myoblasts (20,000 cells/well) were grown in 96-well plates and then differentiated in myotubes as described above. Cell viability was assessed by methylthiazoletetrazolium (MTT) assay. Briefly, myotubes were rinsed with PBS to remove the interfering phenol red providing from DMEM media. Fifty micrograms of MTT in 100 μl of PBS was added to each well for 4 h at 37°C. MTT was then removed, and replaced by 100 μl of DMSO to dissolve the resulting formazan. Absorbance was quantified at 570 nm on micro-plate reader.

### Protein extractions

#### Whole cellular extract

C2C12 myotubes were rinsed three-times in cold PBS. They were then scrapped in cold RIPA lysis buffer (10 mM Tris/HCl, pH 7.4; 150 mM NaCl; 1 mM EDTA; 1% TritonX-100; 0.5% sodium deoxycholate; 0.1% SDS) or in NP-40 buffer (20 mM Tris-base, pH 8.0; 150 mM NaCl; 1% NP-40), both containing anti-proteases (Complete EDTA-free, Roche Diagnostic), anti-phosphatases (Phos-Stop, Roche Diagnostic), and 50 μM PUGNAc [*O*-(2-acetamido-2-deoxy-d-glucopyrano-silidene)amino-*N*-phenyl-carbamate, Sigma]. Proteins extracts were rapidly sonicated using ultra-sonic cell disruptor, and then homogenized with gentle agitation for 1 h at 4°C. Protein estimation of these whole cellular extracts was done using Bradford assay (Biorad).

#### Protein subfractionation

In some cases, proteins extracts were subfractionated as previously described (27). Briefly, myotubes were scrapped in cold cytosol lysis buffer (50 mM Tris/HCl, pH 7.5; 5 mM EGTA; 2 mM EDTA; 5 mM DTT; 0.05% digitonin) containing the inhibitors as described just above. Cell lysates were then centrifuged at 9,500 rpm for 30 min at 4°C, and supernatants (corresponding to the cytosol-enriched fraction) were carefully removed while the pellets (included the membrane-enriched fraction and the myofilament fraction) were discarded. The protein content of the cytosol-enriched fraction was assayed using the Bradford’s method.

### Protein desalting

#### Chloroform/methanol precipitation

Briefly, the sample volume was adjust to 200 μl. Six hundred microliters of methanol were added and sample was vortexed. After addition of chloroform (150 μl volume) and a brief vortex, 400 μl of water were finally added, and sample was briefly vortexed. After centrifugation at 13,000 rpm for 5 min, the upper aqueous phase was carefully discarded while the interface layer (corresponded to proteins precipitate) was leaved intact. A volume of 450 μl of methanol was added, then the sample vortexed before being centrifuged at 13,000 rpm for 5 min. The supernatant was removed and the pellet dried. To ensure the later resolubilization of the pellet, the use of speed-vacuum was avoided, and the sample was air-dried for few minutes.

#### Zeba spin column

In some cases, desalting was performed using the Zeba Spin Desalting Column (Thermo Scientific) with a cut-off of 7 kDa. Column was prepared by a simple centrifugation at 1500 g for 1 min, followed by the deposit of proteins sample to the top of the resin. A stacker of ultrapure water was applied to ensure maximal protein recovery.

### Enzymatic and chemical deglycosylation

Before deglycosylation reaction being performed, samples were desalted using the Zeba Spin Column. Each reaction was performed on 200 μg of proteins from whole cellular extract as well as on proteins from cytosol-enriched fraction. Anti-proteases were added to reaction mixtures. Quantity and incubation time were determined for each deglycosylation protocol to ensure a total deglycosylation. Once reaction was achieved, proteins were precipitated using the chloroform/methanol protocol as described above for each deglycosylation protocol.

#### Peptide: *N*-glycosidase F (PNGase F) deglycosylation

Proteins were denatured by boiling 10 min in SDS-containing denaturing buffer (10× Glycoprotein Denaturing Buffer, New England Biolabs, NEB). SDS, which could lead to an inhibition of glycosidase activity, was then neutralized by adding NP-40 (10% NP40 buffer, NEB). The 10× G7 reaction buffer (NEB) was finally diluted to a final concentration of 1×. Five hundred units of PNGase F (NEB) were added, and the reaction mix was incubated for 4 h at 37°C.

#### Beta-elimination

The GlycoProfile™β-Elimination kit (Sigma-Aldrich) was used to release *O*-glycans from proteins. It removes efficiently and specifically O-linked carbohydrates from glycoproteins without protein degradation (28), permitting consequently downstream proteomics analyses. The β-elimination reagent mixture was prepared as described by the manufacturer, and added equal to 20% of the sample volume. Incubation was performed at 4°C for 8 h. This incubation time was chosen since shorter reaction time was insufficient to be efficient on deglycosylation, whereas longer reaction time (or incubation at room temperature) leads to a slight protein degradation observed by SDS-PAGE profile (data not shown), which is incompatible with the downstream proteins analysis. Once reaction achieved, 1 M HCl was added to bring pH to 6–8.

#### β-*N*-acetyl-hexosaminidase

The removal of β-linked *N*-acetyl-hexosamine was performed enzymatically using the β-*N*-acetyl-hexosaminidase (NEB). This enzyme was chosen since it did not lead to protein degradation as we observed for enzymes from other manufacturers. The G2 buffer (NEB) was added to proteins sample, as well as 50 U of enzyme. Reaction was performed overnight at 37°C.

### Proteins labeling

#### Labeling and/or coupling of *O*-GlcNAcylated proteins

##### Galactosyltransferase labeling

Two hundred micrograms of proteins were chloroform/methanol precipitated. Resulting pellet was resuspended in 20 mM HEPES, pH 7.9 added with 1% SDS, and heated at 90°C for 10 min. Sample was then homogenized at room temperature to ensure the solubilization of proteins. To label *O*-GlcNAcylated proteins with GalNAz, the Click-iT™*O*-GlcNAc Enzymatic Labeling System was used (Molecular Probes). Briefly, Gal-T1 (Y289L) was incubated with proteins in labeling buffer (containing 20 mM HEPES, pH 7.9; 50 mM NaCl; 2% NP-40; 5.5 mM MnCl_2_; 25 μM UDP-GalNAz), according to manufacturer’s recommendations. Reaction was performed at 4°C under gentle agitation for 20 h. All reagents were provided in the kit. Once labeling achieved, proteins were chloroform/methanol precipitated.

Note that the volume of each reagent was adjusted for higher proteins quantity, i.e., when 500 μg of proteins were labeled. This quantity of proteins was suitable to detect with a good sensitivity the cytosolic *O*-GlcNAcome on 2D-gel, according to preliminary experiments.

##### Fluorophore coupling

All coupling reactions were performed using commercially available kits on proteins metabolically labeled with GlcNAz, or on proteins enzymatically labeled with GalNAz. Briefly, proteins pellet was resuspended in 50 mM Tris/HCl, pH 8.0, 1% SDS. The Click-It Reaction Buffer containing the fluorophore was added, followed by CuSO_4_ (2 mM final concentration) and Click-iT™ Reaction Buffer Additives 1 then 2. Incubation was performed under rotation end-over-end for 20 min in dark and at room temperature. Once coupling performed, sample was chloroform/methanol precipitated. A second step of methanol wash was added to remove the residual reaction components. The pellet was heated at 70°C in Laemmli buffer for monodimensional gel electrophoresis, or diluted in solubilization buffer, heated at 37°C for 10 min for bidimensional gel electrophoresis.

When the coupling was performed with TAMRA, the Click-iT™ Protein Analysis Detection Kit was used (Molecular Probes). When coupling was done with Alexa Fluor^®^ 488, the Click-iT^®^ Protein Reaction Buffer Kit, and the Alexa Fluor^®^ 488 alkyne (Molecular Probes) were used. Note that Alexa Fluor^®^ 488 alkyne was diluted in DMSO at a concentration of 4 mM, for a final concentration of 40 μM in the Click-It Reaction Buffer.

#### Labeling of whole proteins

The whole proteins pattern was labeled using the T-Red 310 fluorescent chromophore (T-Dye Series, NH DyeAGNOSTICS). Briefly, 100 μg of proteins were desalting using Zeba Spin Column. The T-Dye, diluted in T-Dye solvent, was added to proteins sample, and then incubated for 30 min on ice. The proteins label with T-Red 310 were then mixed with 400 μg of non-labeled proteins from the same biological sample, and submitted to chloroform/methanol precipitation.

### Electrophoresis

#### Monodimensional gel electrophoresis

Proteins extracted from myotubes were boiled for 5 min in Laemmli buffer (62.5 mM Tris/HCl, pH 6.8; 10% glycerol; 2% SDS; 5% β-mercaptoethanol; 0.02% bromophenol blue) and resolved by SDS-PAGE. Proteins were separated by 7.5% acrylamide:bisacrylamide [(37.5:1), Biorad] SDS-PAGE. Image acquisitions were done with the ChemiDoc MP Imager, a CCD imager, using the Image Lab 4.0.1 software (Biorad).

#### Bidimensional gel electrophoresis

##### Isoelectrofocalisation

Five hundred micrograms of proteins from cytosolic-enriched fraction were precipitated using the chloroform/methanol protocol. The pellet were resolubilized in rehydration buffer (7 M urea, 2 M thiourea, 100 mM dithiothreitol, 4% CHAPS, 4% ASB-14, 1% IPG buffer pH 3–10 non-linear, 0.002% bromophenol blue). To ensure a total resolubilization of the proteins pellet, sample was incubated at 37°C for 5–10 min, following by homogenization under vigorous agitation at room temperature for 1 h. The sample was applied on a pre-cast immobilized pH gradient (IPG) strips (18 cm, pH 3–10, non-linear, GE Healthcare Life Science). Complete sample uptake was carried out overnight for a passive rehydration at room temperature. Focusing was carried out at 20°C under a current limit of 50 μA per strip on PROTEAN^®^ i12 isoelectrofocalisation (IEF) cell (Biorad), and performed at 50 V for 5 h (active rehydration step), 250 V for 1 h (fast progression), followed by a ramping to 10,000 V for 4 h (gradual progression), and was completed at 10,000 V (fast progression) for a total of 60,000 V/h.

##### Second dimension

After isoelectric focusing, the IPG strips were equilibrated for 20 min at room temperature under gentle agitation in reducing solution [6 M urea; 0.375 M Tris/HCl, pH 8.8; 30% glycerol (v/v); 2% SDS (w/v); 2% DTT (w/v)]. They were then equilibrated for a further 20 min in an alkylating solution, which was identical to the reducing solution except that the DTT was replaced by 2.5% (w/v) iodoacetamide. The equilibrated IPG gels were applied to the top of a 8.5% StrenghtAcryl™(Proteomic Solutions) gel and sealed with concentrating acrylamide gel. Electrophoresis was carried out at 10°C with the Protean II XL Cell (Biorad) in running buffer (0.02 M Tris-base, 0.2 M training ion, 0.1% SDS) at 60 V for 22 h. Training ion was glycine for lower buffer, and tricine for upper buffer.

### Proteins visualization

#### Fluorescence detection

Once electrophoretic separation achieved, gels were rinsed in ultrapure water to remove the excess of SDS. Gels were immediately scanned using the Chemidoc MP imager (Biorad) for monodimensional gels or with Typhoon 9400 (GE Healthcare) for 2D-gels.

##### Chemidoc MP acquisitions

Image capture of monodimensional gels images was performed using Chemidoc MP imager under Image Lab™ software. Detection of fluorophores was done with epi-illumination blue, green, or red, in combination with emission filter of 530 BP (band pass) 30, 605 BP 50, and 695 BP 55 nm, for detection of Alexa Fluor^®^ 488, ProQ Diamond, and Sypro Ruby, respectively. The exposure times were chosen to obtain the higher signal/background ratio without a saturation of signals.

##### Typhoon 9400 acquisitions

Images acquisition on Typhoon 9400 was performed with the Typhoon Control Software. The detection of fluorophores was done with blue, green, or red excitation lasers (wavelength of 488, 532, or 633 nm), in combination with emission filters of 520 BP 40, 580 BP 30, 670 BP 30, or 610 BP 30 for detection of Alexa Fluor^®^ 488, ProQ Diamond, T-red 310, or Sypro Ruby. To set PMT (Photo Multiplier Tube) gain, acquisition was performed with low resolution (1,000 μm for pixel size) under normal sensitivity. The PMT was progressively increased until the signals were saturated. The pixel values were determined with ImageQuant Software, if they were comprised between 1 and 100,000, signals were not saturated. When optimal PMT determined (between 600 and 850 V according to fluorophore), acquisition was done under high sensitivity with 200 μm resolution.

#### Proteins staining

After visualization of fluorescent proteins, gels were fixed in methanol (50%, v/v)/ATCA (10%, w/v) at least for 1 h 30 min. Gels were then rinsed with ultrapure water, 6 min × 10 min.

##### ProQ diamond staining

Gels were stained using ProQ Diamond (Molecular Probes, Invitrogen) to detect the phosphoproteome. Incubation was performed overnight under gentle agitation. To avoid background, gels were extensively destained in 50 mM sodium acetate, pH 4.0, containing 15% 1,2-propanediol (29), at least 6 h × 1 h or beyond when background is high. After several washes with ultrapure water, gels were scanned on Typhoon 9400 or on Chemidoc MP. All steps were performed in dark, and gels were immediately scanned after the end of the protocol. To control the specificity of staining, negative control experiments were done on proteins dephosphorylated prior to their electrophoretic separation. All staining and destaining steps were optimized according to the detection of Peppermint Stick markers (Molecular Probes).

##### Sypro ruby staining

Total levels of proteins were revealed on the same gels using Sypro Ruby (Molecular Probes, Invitrogen) staining. Gels were incubated in staining solution overnight at room temperature under gentle agitation. Gels were successively washed once with ultrapure water, twice in destaining solution [10% methanol (v/v), 7% acetic acid (v/v)], and finally twice with ultrapure water, each bath with a duration of 10 min. All steps were performed in dark, and gels were immediately scanned after the end of the protocol.

## Results

### Comparison of the labeling with TAMRA or Alexa Fluor^®^ 488

**Figure 1:**
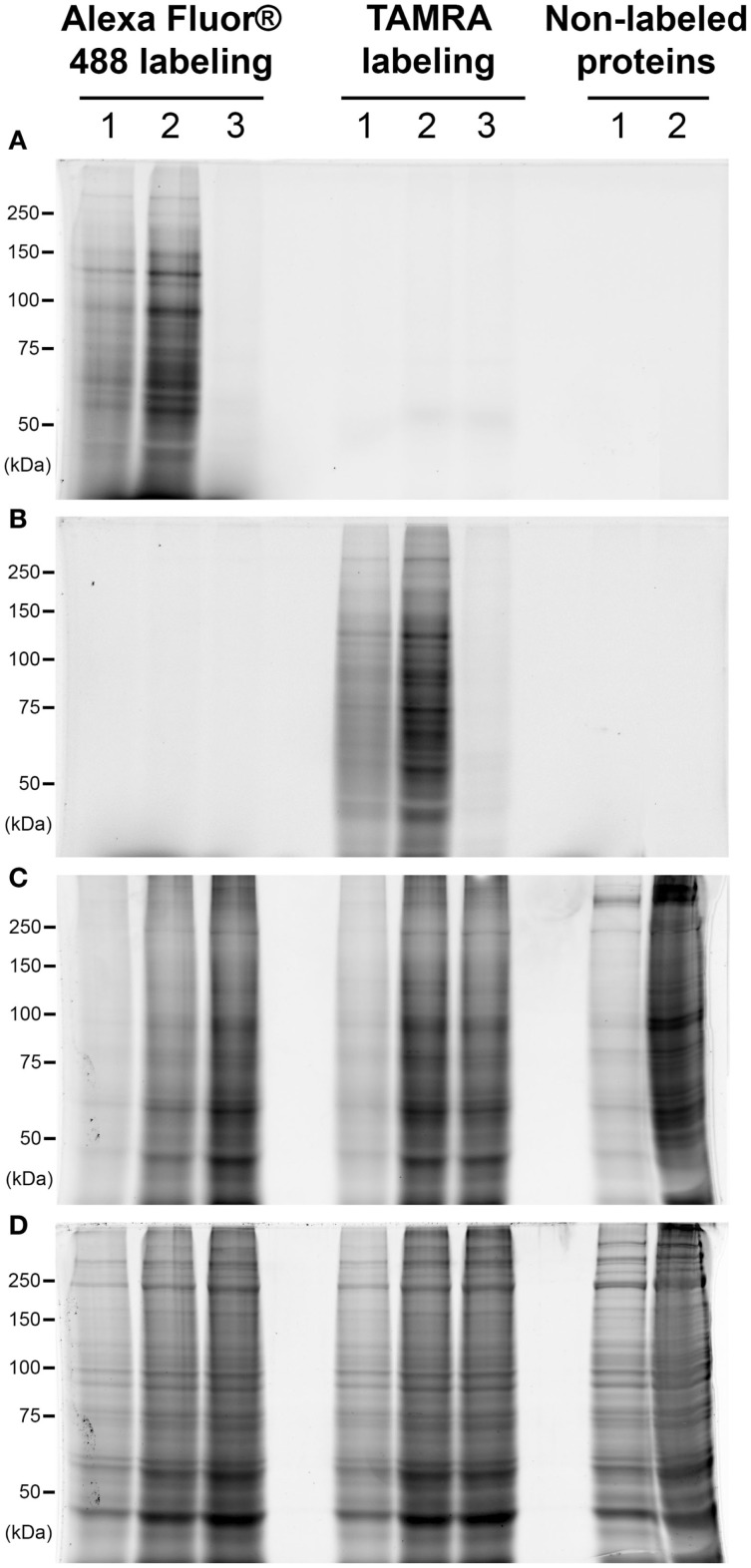
**Comparison of the labeling with TAMRA or Alexa Fluor^^®^^ 488**. Proteins corresponded to 25 or 100 μg of whole cellular extract (lanes 1 and 2, respectively) were labeled with galactosyltransferase and coupled with two different fluorophores: Alexa Fluor^®^ 488 or TAMRA. In each case, the same protocol was applied on 100 μg of proteins but omitting the galactosyltransferase, corresponding so to non-labeled proteins (lane 3). Proteins were then separated on a 7.5% SDS-PAGE, and fluorophores detection was done at the end of the electrophoresis. Gel was then fixed and successively stained with the ProQ Diamond and Sypro Ruby. Images acquisition was performed sequentially after each staining. All acquisitions were done using the Chemidoc MP imager. **(A)** Blue epi-illumination was applied on the gel to detect the Alexa Fluor^®^ 488-labeled proteins. **(B)** Green epi-illumination was applied on the gel to detect the TAMRA-labeled proteins. **(C)** Green epi-illumination was applied on the gel to detect the phosphorylated proteins after the ProQ Diamond staining. **(D)** Red epi-illumination was applied on the gel to detect the whole proteins extract after the Sypro Ruby staining.

We have firstly compared the signals obtained after labeling of *O*-GlcNAcylated proteins with two different fluorophores: the TAMRA or the Alexa Fluor^®^ 488. Data were presented on Figure 1. Briefly, 25 or 100 μg of proteins (lanes 1 and 2, respectively) corresponding to whole proteins extract were labeled using galactosyltransferase in order to add a residue of azido-modified *N*-acetylgalactosamine on GlcNAc moieties. Through the Click chemistry, the azide group was coupled to an alkyne-modified fluorophore. Proteins were separated by SDS-PAGE, and fluorescent proteins were detected immediately after electrophoresis. As shown on Figure 1A, blue epi-illumination lead to the detection of Alexa Fluor^®^ 488-labeled proteins, the intensity of signals being proportional to the amount of proteins per lane. As expected, TAMRA-labeled proteins, as well as non-labeled proteins, were not detected using blue epi-illumination. A green epi-illumination was used to detect the TAMRA-labeled proteins (Figure 1B). Similarly to the Alexa Fluor^®^488-labeled proteins, the intensity of signals was proportional to the quantity of proteins. Nevertheless, the signals were a little more intense for Alexa Fluor^®^488 labeling than TAMRA-labeling; in addition, the TAMRA signals were a little less defined than those obtained after Alexa Fluor^®^488 labeling. In both cases, when galactosyltransferase was omitted from reaction buffer, we did not observe any signal in blue nor green epi-illumination, demonstrating the specificity of fluorophore coupling (lanes 3). Note that the proteins patterns were identical in both cases (lanes 1 or 2).

After the detection of *O*-GlcNAcylated proteins, gels were fixed, then rinsed, and scanned again with blue and green epi-illuminations. We observed a slight decrease in the intensity of signals (data not shown), so that the detection of the fluorescent *O*-GlcNAcylated proteins should be done immediately after electrophoresis. The phosphoproteome was then detected after the ProQ Diamond staining as shown on Figure 1C. The proteins patterns were identical for Alexa Fluor^®^ 488- or TAMRA-labeled proteins as well as for the non-labeled proteins, suggesting that the fluorophores did not affect the ProQ Diamond staining. The TAMRA fluorophore and the ProQ Diamond staining were detected with green epi-illumination, thus, as expected in view of excitation and emission wavelengths, the TAMRA labeling was not compatible with the downstream detection of phosphorylated proteins with ProQ Diamond staining. In contrast, the Alexa Fluor^®^ 488-labeled proteins were detected in the same extent than the post-fixation detection of proteins (data not shown).

The proteins were finally stained with Sypro Ruby, leading so to the detection of whole proteome (Figure 1D). The proteins profiles were identical for labeled proteins (with both fluorophores) as well as non-labeled proteins. In view of these data, the Alexa Fluor^®^ 488 labeling was preferred to TAMRA labeling in all the following experiments.

### Comparison of GlcNAz metabolic incorporation versus galactosyltransferase labeling after N- or O-deglycosylation

**Figure 2:**
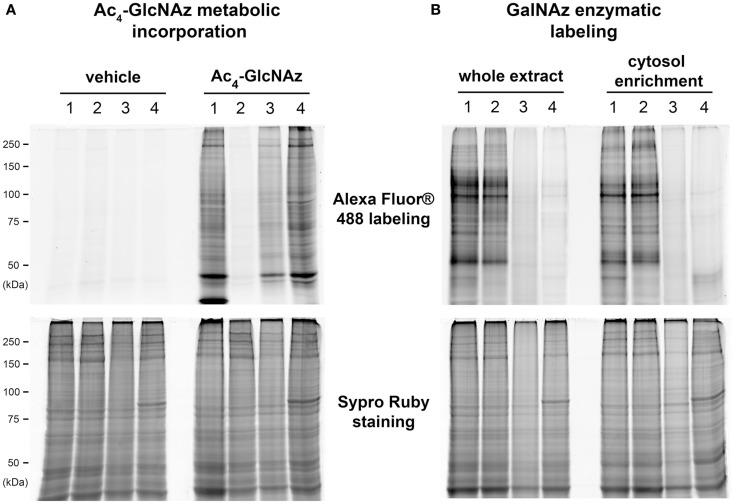
**Comparison of GlcNAz metabolic incorporation versus the galactosyltransferase labeling after N- or O-deglycosylation**. A 100 μg of proteins corresponding to whole proteins extract or to cytosol-enriched fraction were labeled with Alexa Fluor^®^ 488 after metabolic incorporation of Ac_4_-GlcNAz **(A)** or enzymatic labeling with galactosyltransferase **(B)**. After electrophoresis, blue epi-illumination was applied on gels to detect the Alexa Fluor^®^ 488-labeled proteins. Gels were fixed and stained with Sypro Ruby; red epi-illumination permitted the detection of the whole pattern of proteins or cytosol-enriched proteome. Proteins were deglycosylated prior to the labeling with fluorophore. (1) Corresponded to control conditions, i.e., the non-deglycosylated proteins; (2) to *N*-deglycosylated proteins using peptide:N-glycosidase F; (3) to chemically *O*-deglycosylated proteins after β-elimination; and (4) to enzymatically deglycosylated proteins by β-*N*-acetyl-hexosaminidase.

We compared the metabolic and the enzymatic incorporation of azide function on whole cellular extract. All results were presented on Figure 2, those concerning the cytosol-enriched fraction were presented only for GalNAz enzymatic labeling. Indeed, the buffer we used for subfractionation contained dithiothreitol, which could reduce the azide function and therefore interfere with the downstream coupling of alkyne-modified fluorophore in the case of the metabolic incorporation of GlcNAz. We also compared the pattern of labeled proteins after N- or O-deglycosylation.

All results obtained for Ac_4_-GlcNAz metabolic incorporation were presented on Figure 2A. Lane 1 corresponded to non-deglycosylated proteins pattern. When proteins were *N*-deglycosylated with PNGase F prior to the coupling of Alexa Fluor^®^ 488 coupling, we observed a drastic loss of signals (Figure 2A, lane 2). The loss of signals was also observed after chemical O-deglycosylation through β-elimination (Figure 2A, lane 3), but in a lesser extent. In contrast, when deglycosylation was done by β-*N*-acetyl-hexosaminidase, proteins profile was quite similar to non-deglycosylated proteins profile (Figure 2A, lane 4). All together, these data suggested that the Ac_4_-GlcNAz was preferentially incorporated in complex *N*- and *O*-glycans rather than in *O*-GlcNAcylated proteins. It is noteworthy that no signal was observed when C2C12 myotubes were cultured with vehicle, i.e., DMSO, whereas proteins profiles corresponding to Sypro Ruby staining were identical in vehicle or Ac_4_-GlcNAz culture conditions.

Results corresponding to the GalNAz enzymatic labeling were presented on Figure 2B, for whole proteins extract and for cytosol-enriched fraction. As previously, lane 1 corresponded to non-deglycosylated proteins. These profiles were totally different to that obtained after metabolic incorporation of GlcNAz. In contrast, the two profiles (non-deglycosylated whole proteins and cytosolic proteins) were quite similar. The proteins profiles of *N*-deglycosylated proteins were identical to the non-deglycosylated profile (lane 2 compared with lane 1), for whole cellular extract as well as for cytosolic fraction. In contrast, we observed the total loss of signal after chemical and enzymatic O-deglycosylation (lanes 3 and 4, respectively). All together, these data strongly suggested that proteins labeled with the use of galactosyltransferase corresponded exclusively to *O*-GlcNAcylated proteins. This strategy was chosen for the following experiments.

### 2D-gel of *O*-GlcNAcome, phosphoproteome, and whole proteome

**Figure 3:**
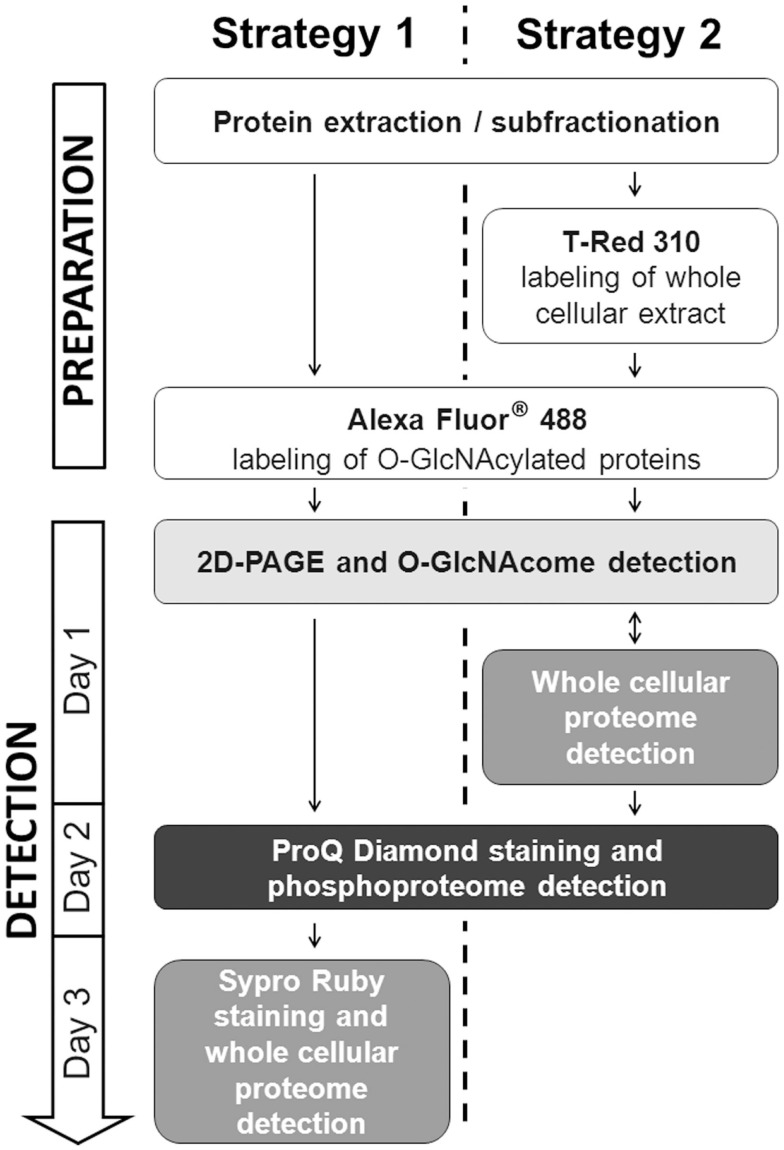
**Schematic representation of the two alternatives for in-gel detection of *O*-GlcNAcome, phosphoproteome, and whole proteome**. The two strategies were indicated in parallel. Note that several experimental steps are common between the two strategies.

**Figure 4:**
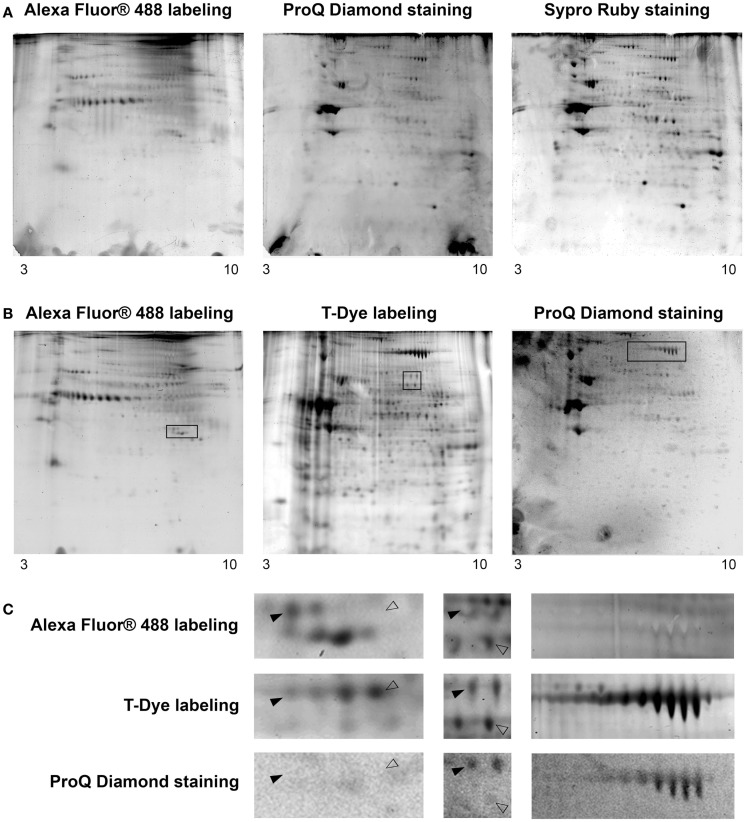
**Cartographies of the *O*-GlcNAcome, the phosphoproteome, and the whole proteome within the same gel**. Five hundred micrograms of cytosol-enriched extract were resolved on bidimensional gel electrophoresis; isoelectrofocalisation was done on non-linear 3–10 IPG dry strip, while second dimension was performed on 8.5% resolving gel. The *O*-GlcNAcome was detected through Click chemistry and Alexa Fluor^®^ 488. The phosphoproteome was imaged after ProQ Diamond staining. The whole proteome was visualized through two approaches: the Sypro Ruby staining, or the T-Dye labeling. **(A)** This workflow corresponded to *O*-GlcNAcome imaging, followed by ProQ Diamond and then Sypro Ruby staining. **(B)** This workflow corresponded to *O*-GlcNAcome imaging, in parallel of whole proteome imaging through T-Dye labeling; the phosphoproteome was then investigated using the ProQ Diamond staining. **(C)** Zoom of zones of interest squared on gels from previous panel. Plain or blank arrows indicated proteins differentially detected on *O*-GlcNAcome, phosphoproteome, or whole proteome images, corresponding so to proteins bearing or not *O*-GlcNAc and/or phosphate moieties.

To be in adequacy with the multiplexed proteomic strategy, we compared two workflows permitting the sequential visualization of the *O*-GlcNAcome, the phosphoproteome, and the whole proteome, as presented in Figure 3. Briefly, in the first strategy, the cytosolic *O*-GlcNAcome was detected through the Click chemistry (labeling of *O*-GlcNAcylated proteins with galactosyltransferase and coupling with Alexa Fluor^®^ 488), followed by the detection of phosphoproteome and the whole cytosolic proteome. The corresponding sequential image acquisition of this strategy was presented on Figure 4A. As shown on this figure, different proteins profiles were obtained from the same gel, corresponding to *O*-GlcNAcome, phosphoproteome, and whole proteome, respectively. It is noteworthy that the image acquisitions were performed on a 3-day period, since the ProQ Diamond and the Sypro Ruby required overnight staining.

The second workflow, described on Figure 3, was done as followed. A double labeling was performed. The *O*-GlcNAcylated proteins were labeled as previously described, while the whole proteins were labeled with the T-Red 310 fluorescent chromophore, a dye having excitation and emission wavelengths compatible with the use of Alexa Fluor^®^ 488 and the ProQ Diamond. We have tested different combination of labeling, i.e., Alexa Fluor^®^ 488 firstly followed by T-Dye labeling, and reciprocally. When the sequence of labeling was Alexa Fluor^®^488 labeling, and then T-dye labeling, we were neither able to obtain a well-resolved image of the *O*-GlcNAcome nor the whole proteome, suggesting that this combination of labeling was problematic and need to be discarded (data not shown).

The second combination of labeling was T-Dye labeling followed by the labeling of *O*-GlcNAcylated proteins as described in the Section “Detailed Experimental Procedures.” After both labeling, proteins were resolved on bidimensional gel electrophoresis, and images were sequentially acquired according to excitation and emission wavelengths of both dyes. After images acquisition, the ProQ Diamond staining was done to imaging the phosphoproteome. All these results were presented on Figure 4B, in the same order than the sequential image acquisition. It is noteworthy that this strategy required only 2 days for all the workflow. On this figure were indicated three squared zones (one squared zone per gel), each being zoomed on Figure 4C. On this panel, plain arrows and blank arrows indicated spots detected on whole proteome pattern, but detected or not on *O*-GlcNAcome and phosphoproteome images. Thus, proteins spots could be detected on phosphoproteome pattern but not on *O*-GlcNAcome pattern, or vice-versa. We could hypothesized that these spots could correspond to phosphorylated but non-glycosylated proteins, and reciprocally. Of course, we can not exclude that the signals obtained after ProQ Diamond staining or after *O*-GlcNAcylated proteins labeling were under the detection threshold. In some case, spots were detected in all images, suggesting that the corresponding proteins could bear simultaneously *O*-GlcNAc and phosphate moieties. It should be mentioned that the same data were obtained from each zone of interest in the first strategy, but only results corresponding to the second strategy were included in Figure 4.

## Discussion

Proteomics community celebrates the 20 years old of the term proteomic, proposed to the Sienna conference in 1994 (30). Historically, bidimensional gel electrophoresis was largely used in proteomic approaches. However, 15 years ago, the benefits/advantages of bidimensional gel electrophoresis approach were questioned. In fact, as pointed out by Fey and Larsen (31), 2D gel requires manual dexterity and precision to reproduce precisely and is thus not well-suited as a high-throughput technology. However, despite these drawbacks, 2D-electrophoresis offers a resolution and sensitivity, which are exquisite and unsurpassed if one wants a global view of “cellular activity” (31). Nevertheless, though sometimes criticized, bidimensional gel electrophoresis remains one of the most widespread techniques in the field of functional proteomics. Through proteomes comparison between cells, tissues, or organs, providing from different physiological or pathological conditions, 2D-electrophoresis allowed a proteome map at a given time, and offered a large-scale analysis about the alterations occurring in protein expression levels and modifications. Moreover, consecutively to the numerous approaches used by the proteomic community, the advantages and the drawbacks of gel-based proteomic methods, in particular, 2D-electrophoresis, are well-known nowadays, and should be considered when proteomic approach is initiated (32, 33). Since this kind of approach could be done without the need of high-sophisticated equipment (and so accessible to a large panel of laboratories), we proposed in this paper, a method based on 2D-gel electrophoresis, to detect in a same gel the *O*-GlcNAcome, the phosphoproteome (and therefore the interplay between both post-translational modifications), and the whole cellular proteome.

The use of fluorescence detection in 2D-differential gel electrophoresis had substantially upgraded the potential and the power of bidimensional electrophoresis for the analysis of protein expression differences, and for the detection of post-translational modifications. Through multiplexed technologies, reproducibility, robustness, and technical confidence greatly increased for several years (33, 34). This methodology, based on the use of non-overlapped fluorescent dyes such as ProQ Diamond, ProQ Emerald, and Sypro Ruby, allowed the parallel determination of phosphorylation, glycosylation, and whole proteins patterns through the comparison of different images acquired from the same gel (22, 23, 35). However, the ProQ Emerald, while more sensitive than the standard periodic acid–Schiff base method using acidic fuchsin dye, also detected the *N*-glycosylated proteins. Thus, the ProQ Emerald is not the exclusive method for the detection of O-GlcNAcylation. Few years ago has emerged the use of chemical reporters of glycosylation, originally developed by the Bertozzi’s laboratory [Ref. (36); reviewed in Ref. (37)], and azide- or alkyne-bearing analogs of monosaccharide were currently available. In this way, the azido-modified monosaccharide GlcNAz could be used for metabolic incorporation of azide group on *O*-GlcNAcylated proteins. Alternative method of post-lysis was also developed in Hsieh–Wilson’s group, based on the transfer of azido-modified *N*-acetylgalactosamine on *O*-GlcNAc moieties through a mutant galactosyltransferase (38, 39), this advanced chemoenzymatic strategy for proteomic analysis lead to the development of commercially available reagents for fluorescent labeling of *O*-GlcNAcylated proteins (38). These bioorthogonally functionalized proteins extract were then labeled with a probe, permitting downstream the purification or the detection of *O*-GlcNAcylated proteins after Staudinger ligation or copper-catalyzed azide–alkyne cycloaddition (37).

This Click chemistry is nowadays a promising method for detection and/or purification of *O*-GlcNAcylated proteins. This approach is characterized by a relative simple and improved methodology and allowed a good sensitivity, as well as reproducibility. In this paper, we compared both methods (metabolic and enzymatic) of incorporation of azide group on *O*-GlcNAcylated proteins. According to our data, the chemoenzymatic labeling should be preferred for labeling of GlcNAc moieties since this strategy offers a serious specificity of GlcNAc moieties detection after incorporation of the azido-modified *N*-acetylgalactosamine compared with GlcNAz incorporation (in particular, in complex glycans). In addition, the subcellular fractionation remains a helpful device to reduce the contamination of proteins with the glycoproteins bearing complex *N*- or *O*-glycans and enhanced the specificity as well as the sensitivity for *O*-GlcNAcylated proteome analysis.

The major finding in this paper was the detection of *O*-GlcNAcome, phosphoproteome, and whole proteome in only one gel. Briefly, the chemical labeling of *O*-GlcNAc moieties with Alexa Fluor^®^ 488 lead to the detection of the *O*-GlcNAcome, and downstream detection of phosphoproteome was done after ProQ Diamond staining. The global proteome could be detected either Sypro Ruby staining or after covalent labeling of proteins using the T-Dye fluorophore. In both cases, the proteins profiles were similar. In our finding, the double labeling was preferred since the *O*-GlcNAcome and the whole proteome images could be acquired simultaneously. Whatever the approach used, we were able to discriminate unmodified proteins from proteins which were *O*-GlcNAcylated, or phosphorylated or both. It remains important to keep in mind the limitation of this approach, inherent to 2D-gel approach as well as sensitivity of dyes, such as ProQ Diamond staining or the labeling of *O*-GlcNAc proteins, in particular, in view of the detection threshold. In this way, a relative important amount of proteins are necessary to the detection of *O*-GlcNAcome and the phosphoproteome, leading so to a slight decrease in spot resolution of 2D-gel. It should also be mentioned that this method should be optimized according to the studied tissues or the cell lines in terms of the amount of labeled proteins and the electrophoretic conditions. In addition, the effective phosphorylated and/or *O*-GlcNAcylated state of a protein should be attempt in the validation steps.

Indeed, the final purpose of this method was to propose a simple methodology to determine the variation of O-GlcNAcylation, and/or phosphorylation, and/or protein expression. This semi-quantification could be thereafter determined through images analysis using specific proteomic softwares, by comparison of proteins extract, resulting from different physiological or cellular conditions, for example, healthy versus pathological conditions, or untreated versus treated cells, and so on. Differential spots could be then excised from gel to be submitted to proteolytic digestion and mass spectrometry analysis through a bottom-up proteomic approach. All data providing from this kind of approach need to be validated, and suffer from the lack of a precise quantification. In this way, recent developments in mass spectrometry, more particularly with the breakthrough of stable isotope labeling with amino acids in cell culture (SILAC) or the label-free quantification, are nowadays powerful and adaptable tools for quantitative proteomic (5).

To conclude, we propose herein a method for the profiling of *O*-GlcNAcome, phosphoproteome, and whole proteome in a completely blind and global approach. The recent developments render 2D-electrophoresis to be still considered seriously for proteome analysis and to be again one of the preferred methods in many laboratories. This method remains fast, simple, and easy to use, without the need of high-sophisticated equipment, and so accessible to a large panel of laboratories. The major finding was the proof-of-concept of a 2D-gel-based multiplexed strategy, in which three important informations were gained within only one gel. The use and large choice of fluorescent probes enhanced the sensitivity and powerful of this technique and allowed multiplexed proteomic technology to detect O-GlcNAcylation and phosphorylation two key post-translational modifications in the regulation of many cellular processes.

## Conflict of Interest Statement

The authors declare that the research was conducted in the absence of any commercial or financial relationships that could be construed as a potential conflict of interest. The Guest Associate Editor Tony Lefebvre declares that, despite being affiliated at the same institution as all of the authors, the review process was handled objectively and no conflict of interest exists.
